# Morphological and functional effects of graphene on the synthesis of uranium carbide for isotopes production targets

**DOI:** 10.1038/s41598-018-26572-5

**Published:** 2018-05-29

**Authors:** L. Biasetto, S. Corradetti, S. Carturan, R. Eloirdi, P. Amador-Celdran, D. Staicu, O. Dieste Blanco, A. Andrighetto

**Affiliations:** 1 Università di Padova, Dipartimento di Tecnica e Gestione dei Sistemi Industriali, Stradella San Nicola 3, 36100 Vicenza, Italy; 2 INFN-Laboratori Nazionali di Legnaro, Viale dell’Università 2, 35020 Legnaro (PD), Italy; 3 Università di Padova, Dipartimento di Fisica e Astronomia, Via Marzolo 8, I-35131 Padova, Italy; 4 European Commission, Joint Research Centre, Directorate G for Nuclear Safety and Security, Unit G.I.5, Advanced Nuclear Knowledge unit, Postfach 2340, 76215 Karlsruhe, Germany; 5 European Commission, Joint Research Centre, Directorate G for Nuclear Safety and Security, Unit G.I.3, Nuclear Fuel Safety unit, Postfach 2340, 76215 Karlsruhe, Germany

## Abstract

The development of tailored targets for the production of radioactive isotopes represents an active field in nuclear research. Radioactive beams find applications in nuclear medicine, in astrophysics, matter physics and materials science. In this work, we study the use of graphene both as carbon source for UO_2_ carbothermal reduction to produce UC_x_ targets, and also as functional properties booster. At fixed composition, the UC_x_ target grain size, porosity and thermal conductivity represent the three main points that affect the target production efficiency. UC_x_ was synthesized using both graphite and graphene as the source of carbon and the target properties in terms of composition, grain size, porosity, thermal diffusivity and thermal conductivity were studied. The main output of this work is related to the remarkable enhancement achieved in thermal conductivity, which can profitably improve thermal dissipation during operational stages of UC_x_ targets.

## Introduction

Actinide carbides are potential candidates both as materials for improving the safety of generation IV fast nuclear reactors^1,2^, and as target for the production of second generation Isotope Separation On Line facilities (ISOL)^3^.

In both cases the material must withstand high temperatures (up to 2273 K) and eventually heat-induced stresses. For this reason, heat dissipation is one of the key issues to be complied for the safety assessment of fuels and development of efficient targets. Consequently targets of actinide carbides are often mixed with an excess of carbon in the form of graphite or pyrolytic carbon in order to tailor their thermal properties. In addition, the isotopic species produced in ISOL targets must be able to escape from the target towards the ion source before they decay. This step of isotope extraction is very problematic, since their decay time may be in the order of few ms.

The aim of this work is the production of targets, for second generation ISOL facilities^4–9^ that possess enhanced thermal conductivity. ISOL targets are generally produced via carbo-thermal reaction of UO_2_ with a carbon source. Beside graphite, different carbon source can be used, e.g. multiwall carbon nanotubes (MWCNTs)^10^ and carbon black^11^. The presence of a certain amount of porosity within the target and controlled grain size^11^ must be preserved to allow the produced isotopes to escape from the target via diffusion-effusion processes^12^.

Actinide carbides differ from transition metal carbides, due to unfilled 5 f orbital in their electronic structure. For instance this leads to strong chemisorption of UC_2_ in graphene^13^ with concomitant consequences in terms of their mechanical and thermal properties.

The thermal conductivity of UC_2_ and UC increases with increasing temperature and ranges from 10–11 (W/mK) at room temperature to 20–21 (W/mK) at 2573 K, for UC_2_ and reaches even higher thermal conductivity values for UC^14^. Other refractory carbides considered for ISOL facilities include TiC [11] produced as nanostructured target in order to enhance its release efficiency, and lanthanum carbide^15^ for which a slip casting process was developed to obtain refractory carbide-carbon composites.

This research is the second part of a project titled “Study of the use of graphene as source of carbon for Uranium Carbide-Graphene nanocomposites production” and has been completed in the frame of Infrastructure/user access at the Joint Research Centre Karlsruhe, Directorate G for Nuclear Safety and Security. Previous studies^16^ have shown how the use of graphene as a carbon source for the carburization of La_2_O_3_ to give LaC_2_ acts as sintering aid. Here the use of graphene as a carbon source for the carburization of UO_2_ was studied. Thermal properties of the obtained graphene-derived carbides (diffusivity, conductivity) were compared to graphite derived UC_2_, to verify if the use of graphene can be beneficial in terms of fundamental properties related to on-line operation of the target materials.

## Results

The carbothermal reaction to obtain the composite disks has been carried out according to the following reaction:

1 $${{\rm{UO}}}_{2}({\rm{s}})+6{\rm{C}}({\rm{s}})\to {{\rm{UC}}}_{2}({\rm{s}})+\mathrm{2C}({\rm{s}})+2\mathrm{CO}({\rm{g}})$$

Using a molybdenum grid as a crucible, it was observed that the parts directly in contact with the metal showed a higher reactivity than expected, independently on the type of carbon source used for the carbothermal reaction. In commonly adopted procedures to produce ISOL targets, the reaction is performed by heat treatment, with the samples in a vertical position, without interaction with metal crucibles being only in contact with a graphite button or disk at their periphery^17^. For this reason, the characterization results reported here refer only to the sections of the samples that were not in contact with molybdenum: Thus they are fully representative of actual ISOL target materials.

### Structural and chemico-physical properties

Table 1 provides an overview of graphite and graphene-derived uranium carbide samples (UC_x_-graphene and UC_x_-graphite) properties before and after the thermal treatment. UC_x_-graphene and UC_x_-graphite have very similar density, and consequently the same level of total porosity, whereas the weight loss observed during synthesis for UC_x_-graphene is slightly higher than that of UC_x_-graphite.

**Table 1:** Composition, shrinkage, weight losses and calculated total porosity of the produced samples.

Sample	Composition [wt.%]			Density [g/cm^3^]	Linear shrinkage [%]	Weight loss [wt.%]	Theoretical weight loss [wt.%]	Total porosity [vol.%]
	UO_2_	Graphite	Graphene					
UC_x_-graphite	79.9	20.1		5.65 ± 0.03	23.1	17.8 ± 1.0	16.6	29.14 ± 0.01
UC_x_-graphene	79.9		20.1	5.53 ± 0.02	18.0	19.2 ± 0.2	16.6	34.90 ± 0.01

Weight losses are higher than those expected theoretically, as previously observed^18^, and this can be ascribed to decomposition of the organic binder used in samples’ preparation and the water desorption from the starting powders, which were not taken into account. Higher shrinkage was observed for UC_x_-graphite.

In Table 2, the results of the carbon content analysis are reported for both the starting carbon sources and the final samples, in which all the carbon (bound to U and free) is taken into account. Moreover, oxygen contents reported in Table 2 were measured on representative samples obtained after heat treatment. Both carbon and oxygen contents are quite similar for both samples, indicating that they followed the similar reaction route in terms of oxide to carbide conversion.

**Table 2:** Carbon and oxygen content analyses.

Sample	No Heat Treatment	After Heat Treatment	
	Carbon content [wt.%]	Carbon content [wt.%]	Oxygen content [wt.%]
Graphite*	96.6 ± 1.8	—	—
Graphene*	99.2 ± 1.8	—	—
UC_x_-graphite	—	15.8 ± 1.1	1.9 ± 0.3^#^
UC_x_-graphene	—	16.6 ± 1.2	2.1 ± 0.2^#^

*after ball milling; ^*#*^*measured on all sample*.

**Figure 1:**
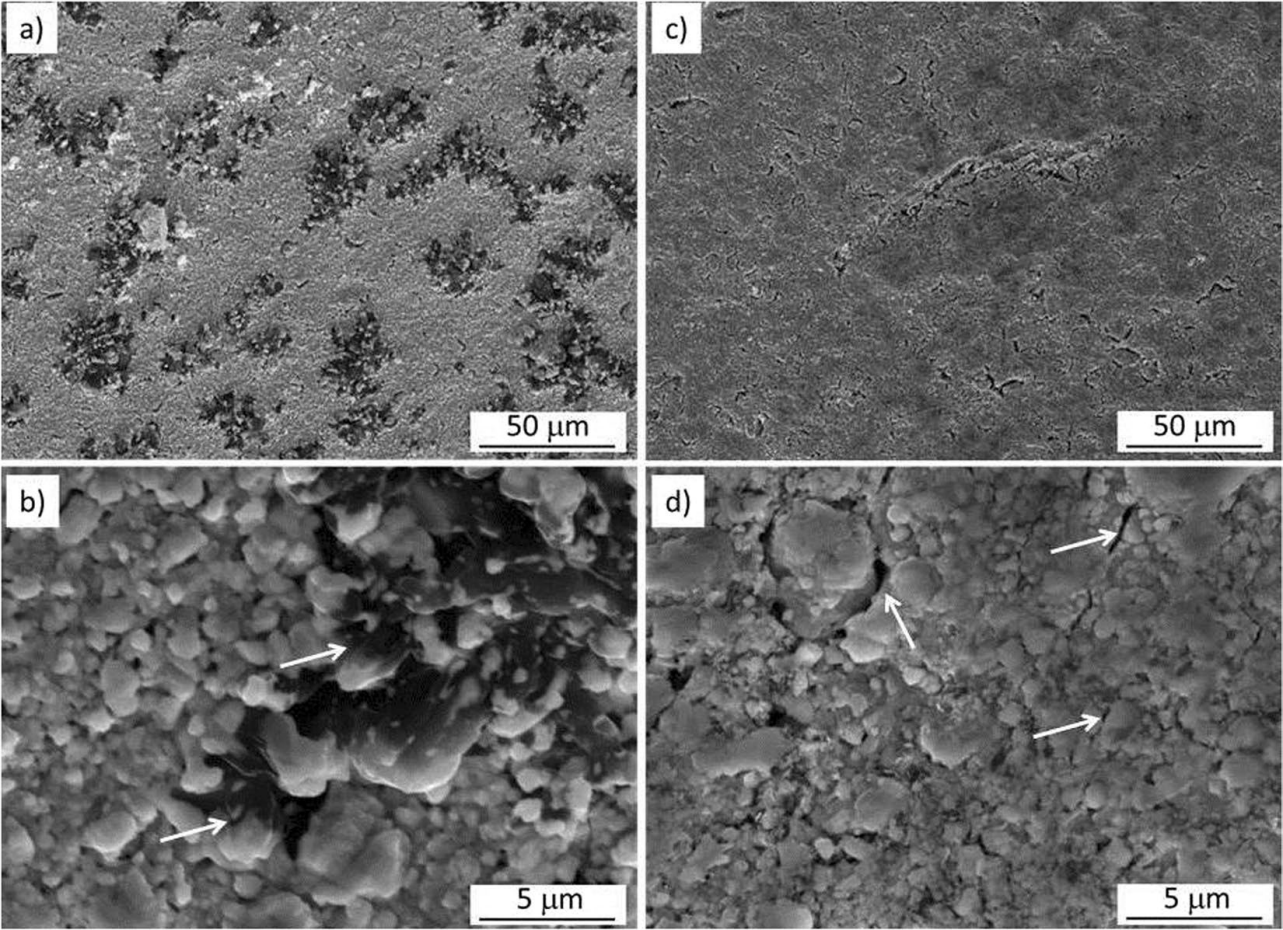
SEM images of UC_x_-graphite (**a,b**) and UC_x_-graphene (**c,d**). Arrows indicate residual carbon in (b) and micro-cracks in (d).

**Figure 2:**
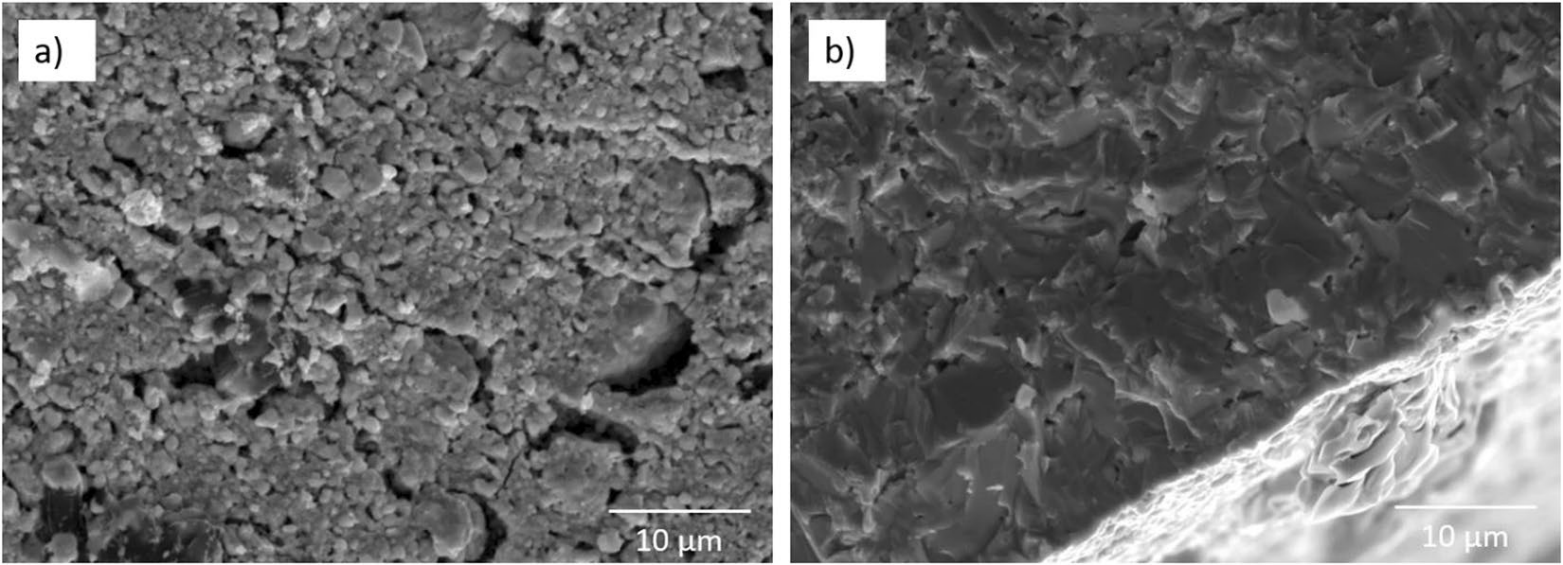
SEM images of the cross-section of UC_x_-graphite (**a**) and UC_x_-graphene (**b**).

The SEM images reported in Fig. 1, show the surface of UC_x_-graphite and UC_x_-graphene and the difference in distribution of the residual carbon in the structure. While carbon is clearly visible for the UC_x_-graphite sample, emerging from the surface as described in an earlier study on UC_x_-graphite^16^, this is not the case of UC_x_-graphene. This latter presents a finer distribution of the residual carbon with local dark areas in the image. Higher magnification images reported in Fig. 1(b,d) show that both structures have a fine distribution of micron-sized uranium carbide grains, locally interrupted by residual carbon in UC_x_-graphite and micro-cracks in UC_x_-graphene with no clear evidence of unreacted carbonaceous phase. The presence of sub-micron sized cracks in UC_x_-graphene cannot be ruled out because of the limited resolution of SEM. These pores may be responsible of the higher total porosity (lower density) of UC_x_-graphene with respect to UC_x_-graphite. The internal microstructure of both samples is revealed in the SEM images of Fig. 2. In agreement with observations made in Fig. 1, the sample of UC_x_-graphene does not show any apparent presence of carbon, indicating also that higher magnification is needed to reveal the residual multilayered graphene.

**Figure 3:**
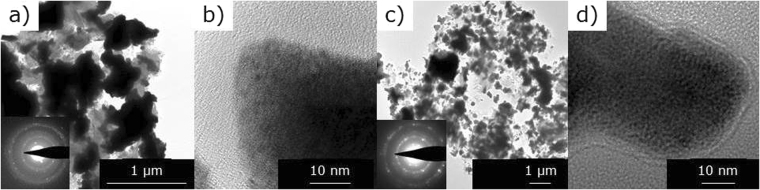
TEM images of UC_x_-graphite (**a,b**) and UC_x_-graphene (**c,d**).

The TEM pictures reported in Fig. 3 show a better distribution of UC_x_-graphene particles (Fig. 3c) compared to UC_x_-graphite (Fig. 3a), where particles are more agglomerated in graphite matrix. The selective area electron diffraction (SAED) pattern of UC_x_-graphite and UC_x_-graphene (insert Fig. 3a,c), show that both samples are polycrystalline. At higher magnification UC_x_-graphene particles (Fig. 3d) are surrounded by a layer which is not visible for UC_x_-graphite (Fig. 3b). The nature of this layer could be linked to the presence and very distribution of graphene present in UC_x_-graphene.

**Figure 4:**
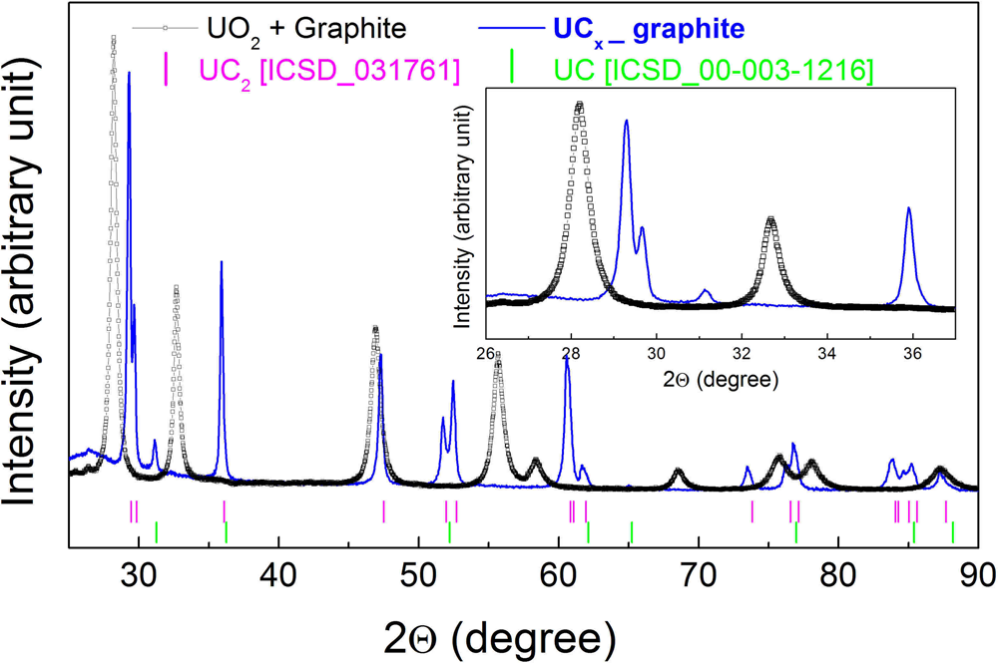
XRD diffraction patterns of UC_x_-graphite before and after the heat treatment at 1973 K under Ar flow during 24 h.

**Figure 5:**
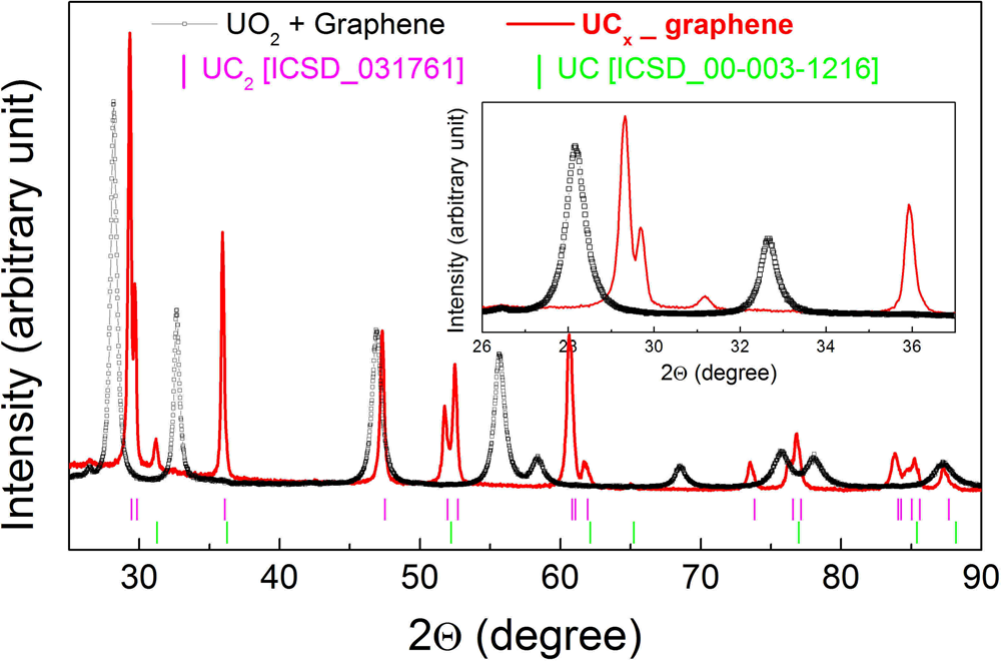
XRD diffraction patterns of UC_x_-graphene before and after the heat treatment at 1973 K under Ar flow during 24 h.

XRD pattern of both samples before (open data points) and after (solid line) the heat treatment are reported in Figs 4 and 5. For both, a complete conversion of the UO_2_ + carbon mixture into uranium carbide is observed. Differently to that expected in eq. (1), a certain amount of UC has been obtained as UC_2_. For both samples, the wt% ratio between UC_2_ and UC, calculated with Rietveld analysis, was 95/5. The presence of UC in the processes designed to obtain UC_2_ by carbothermal reduction of UO_2_ is well documented in literature^18^, and it has been ascribed to the occurrence at the final stages of the synthesis of the following reaction^19,20^.

2 $$3{{\rm{UC}}}_{2}({\rm{s}})+{{\rm{UO}}}_{2}({\rm{s}})\to 4\mathrm{UC}({\rm{s}})+2\mathrm{CO}({\rm{g}})$$

The selected C/UO_2_ starting ratio for the carbothermal reaction enables this mechanism also in the present case, as predicted by the work of Mukerijee^20^. The atmosphere used for the carbothermal reduction (Ar or high vacuum) determines the reaction, due to diffusion of CO by-product, whereas in vacuum the extent of interface between reactants is the controlling step^20,21^. In previous experiments related to composites derived from carbothermal reduction of lanthanum oxide conducted in vacuum, the use of graphene as carbon precursor led to a high degree of sintering, remarkably higher density and low porosity with respect to graphite based composites^14^. In the present case, where the reaction is diffusion controlled, the trend in density and total porosity is opposite, although the difference in porosity between graphite and graphene derived composites is not so remarkable. Furthermore, in a previous work^18^ the total porosity of UC_x_-graphite composites produced in high vacuum starting from UO_2_ powders compacted with graphite in conditions similar to those adopted here was remarkably higher than when the carbothermal reduction was performed under Ar.

Therefore, it can be inferred that the environment where the reduction is conducted is crucial in determining the microstructure of the obtained composites, (i.e. final density and porosity). In the case of a vacuum atmosphere, the presence of graphene is relevant as a sintering aid and the porosity is reduced, as a result of a more efficient surface contact between the reactants. In the case of Ar flow, the fast release of CO while UC_2_ is forming through the highly permeable graphene layers leads to higher porosity than in case of graphite, and is most likely ascribed to carbide grains with size in the nm range, as observed in TEM images. However, further experiments will be performed by conducting the carbothermal reduction of graphene based composites in vacuum, in order to confirm this hypothesis.

In spite of different microstructure, it is worth noting that the reaction goes in both cases to completion, when either graphene or graphite is used as precursor, as demonstrated by XRD analyses, leading to negligible differences in the structure of the produced carbide.

As for the structure of residual carbon in the final bodies, the diffraction patterns and Raman spectra of the very same graphite and graphene used in this work (reported in [16]), show a nanocrystallite size of 44.3 nm for graphite and 15.6 nm for graphene. The presence of residual carbon could not be detected by XRD analysis, either because it is present as an amorphous phase and/or because it is below detection limit or due to the presence of high Z uranium atoms. The crystallite size of UC_2_ in the [112] and [110] directions were calculated and reported in Table 3, showing an asymmetric shape as already reported for lanthanum carbide in [16].

**Table 3:** UC_2_ crystallite size (k = 1).

Sample	UC_2_ crystallite size [112] [nm]	UC_2_ crystallite size [110] [nm]
UC_x_-graphite	74.0	35.0
UC_x_-graphene	48.6	36.3

### Functional properties

**Figure 6:**
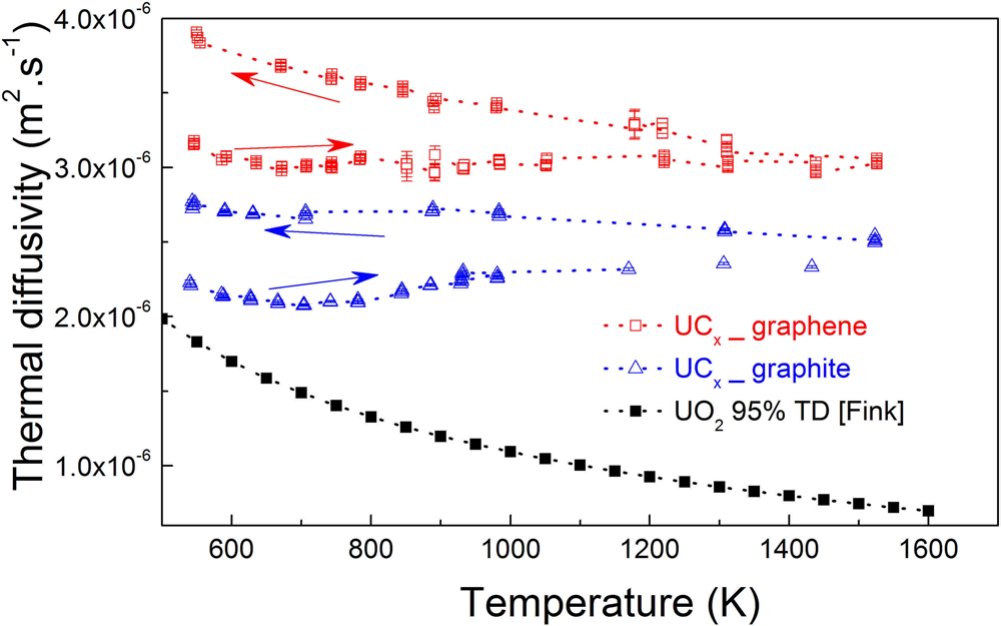
Thermal diffusivity of UC_x_-graphene and UC_x_-graphite.

The thermal diffusivity was measured by the laser flash technique. Thermal diffusivity measurements (Fig. 6) of both final materials up to 1550 K show the beneficial effect of graphene as a starting powder in the capability of the material to dissipate heat. Both samples present remarkable differences between the data obtained in the heating and cooling branches of the measurement. This hysteresis could be caused by structural modifications occurring at high temperature, for example densification by porosity reduction, which could have the effect of increasing diffusivity in the cooling branch of the measurement.

The thermal conductivity *λ* (in W m^−1^K^−1^) was calculated using equation (3) from the measurements of the thermal diffusivity *α* (in m^2 ^s^−1^) and density *ρ* (in kg m^−3^) obtained by tomography and corrected for thermal dilatation using data from^22^, with specific heat *C*_*p*_ (in J kg^−1^ K^−1^) data from^23^.

3 $$\lambda =\alpha \rho {C}_{p}$$

**Figure 7:**
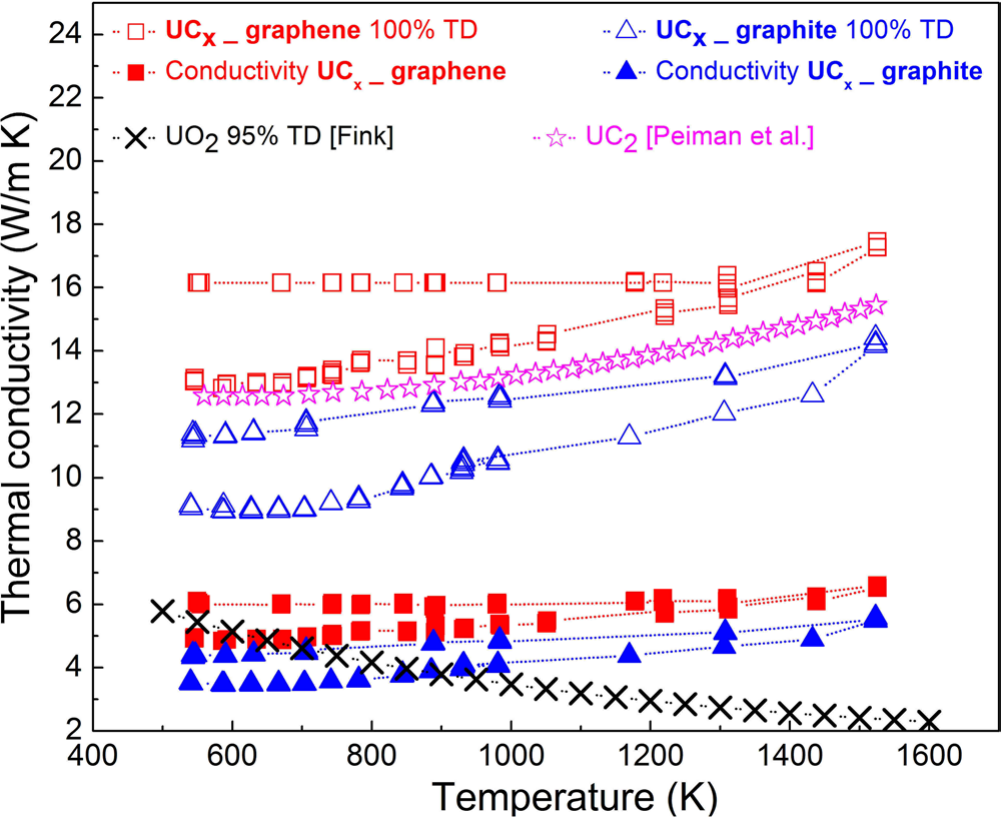
Thermal conductivity measurement of UC_x_-graphene and UC_x_-graphite, compared to estimated values of their corresponding 100% theoretical density (TD).

If only the data obtained in the heating portion of the measurement (right arrows) is, considered an increase of diffusivity by more than 20% is observed in UC_x_-graphene with respect to UC_x_-graphite derived samples. This is confirmed by the derived thermal conductivity shown in Fig. 7, where an increment of thermal conductivity ranging from 20 to 40% was observed for UC_x_-graphene with respect to UC_x_-graphite throughout the observed temperature region (fill data points). Due to the presence of significant porosity, the absolute values of thermal conductivity of both samples resemble those of 95% dense UO_2_^24^ more than UC_2_^14^. For comparison, a projection of thermal conductivity to fully dense UC_x_-graphite and UC_x_-graphene samples was calculated using the Maxwell formula^25^ and is reported (open symbols). It shows, a similar trend to pure, fully dense UC_2_. The trend obtained for UC_x_-graphite is in good agreement with that reported by Greene *et al*.^26^, in which UC_2_-C samples with density of 5 g/cm^3^ showed increasing thermal conductivity with temperature in the range 1873 K–2173 K, with a thermal conductivity of about 5 W/m∙K at 1873 K.

The increase of thermal conductivity in UC_x_-graphene versus UC_x_-graphite is a direct consequence of its intrinsic higher diffusivity, as shown in Fig. 6.

## Discussion

From the chemico-physical and functional characterization reported above, it is clear that graphene derived uranium carbides have higher thermal conductivity than those derived from graphite. This phenomenon is to be ascribed to intrinsic properties of graphene, even if its predictability is not straightforward. The addition of well dispersed graphene platelets on ceramic matrices by powder mixing and sintering has been reported as a method to increase thermal conductivity^27^. In the case of materials obtained by powder pressing, such as silicon nitride, the dispersion of graphene platelets resulted in an anisotropic thermal response, with thermal conductivity increasing in the direction of alignment of the platelets, perpendicular to the pressing axis^28,29^. In this work, however, graphene is not only dispersed in a pressed ceramic matrix but it acts as a carbon source for the reduction of an oxide to a carbide. Considering the hyperstoichiometry of the reaction, the residual graphene in the final samples is either composed of completely unreacted or partially reacted platelets, over which uranium carbide is formed and grown, (cf previously reported carbon nanotubes derived carbides^10^). In apparent contrast to the recently reported results for lanthanum carbide composites derived from graphene^16^, there seems to be no sintering aid effect of dispersed graphene in uranium carbide, as confirmed by bulk density measurements. UC_x_-graphene is indeed characterized by higher porosity. Nitrogen adsorption-desorption analyses could give indications on the presence of micro and mesoporosity, not detectable by SEM, as well as measurement of the true density of sintered UC_x_ could confirm the calculated values of total porosity by eq. 4. As previously described, this behavior can be ascribed to the different reaction environment, i.e, the carbothermal reduction in flowing argon instead of high vacuum leads to a variation in the rate determining mechanisms. In the case of Ar flow atmospheres, CO diffusion is the rate-controlling step, hence the enhanced surface area of multilayered graphene promotes the fast release of gaseous by-products and the final porosity of the composites increases with respect to the graphite-based ones.

## Methods

### Samples preparation

Sample preparation until pressing of the pellet has been done in glove box equipped with a gas MBraun purification system. The atmosphere was kept under nitrogen with a content of H_2_O < 1 ppm and of O_2_ < 50 ppm. UO_2_ sample were prepared by and purchased from the British Nuclear Fuels Ltd. Both Graphite and graphene were used as carbon sources for the synthesis of UC_2_. Graphite powders of mesh size <325 micron were purchased form Sigma-Aldrich. Elicarb^®^ Graphene was purchased from Thomas Swan&Co. Ltd UK. The Elicarb^®^ Graphene powders are high purity platelets of size ranging from 0.5 nm to 3 nm. The reactants were first individually mixed in a planetary ball-mill (3000 rpm and 2 h), Jar and balls were made of ZrO_2_, leading to a powder size <5–10 micron.

The powders were first mixed in an agathe mortar using the hyperstoichiometric ratio reported in reaction (1).

A solution of 2 wt% of phenolic resin in acetone (15 wt%) was added dropwise as binder. The powders were then further mixed in the ball-mill (during 3 h at 3000 rpm).

The powders were then pressed using a uniaxial cold press at 7 ton for 10 min, each. At least five pellets of 13 mm diameter and about 1 mm thickness were prepared for each batch (UO_2_-Graphite and UO_2_-Graphene). The samples were heat threated in one batch under Argon flow in a (Degussa Type VSL) annealing furnace using a Molybdenum crucible at 1963 K for 24 h, heating rate 2 K/min. A Residual Gas Analyzer (Siemens Ultramat 6) was used during the heat treatment in order to monitor the CO evolution and consequently the effect of carbothermal reaction. The weight loss after thermal treatment was measured by an analytical balance Mettler Toledo Mod. SAG204.

### Chemical analysis

Chemical analyses of carbon and oxygen contents have been performed on powdered samples by direct combustion using the infrared absorption detection technique with an ELTRA CS-800 instrument.

### Samples physical characterization

Samples bulk density ρ_bulk_ was measured by weight over volume ratio, and by measuring the mass and the volume using a X-ray Tomography (Nikon XTH 225 Industrial CT scanning device, equipped with a 225 kV microfocus X-ray source with a 3 μm focal spot size). The total porosity of the samples was calculated by:

4 $${{\rm{P}}}_{{\rm{tot}}}=1-{{\rm{\rho }}}_{{\rm{bulk}}}/{{\rm{\rho }}}_{{\rm{th}}}$$

where ρ_th_ is the theoretical density of the final sample, calculated by the mixture rule taking into account the volumetric fractions of UC_2_ and free carbon present in the final samples (eq. 1) and their theoretical solid densities, 11.2 g/cm^3^ for UC_2_ [18], 1.9 g/cm^3^ for graphite and 2.3 g/cm^3^ for graphene, as reported in the suppliers datasheets. The effect of UC presence in the final material was not taken into account in the theoretical density calculation, as it is only 5 wt% of the UC_2_/UC mixture, as reported in the results section, and identical between UC_x_-graphite and UC_x_-graphene. The shrinkage percentage was determined considering only the average thickness of the pellet measured with an absolute Digimatic Indicator ID-S (Mitutoyo), before and after heat treatment.

Scanning electron microscopy was performed on a Philips XL 40 using a tungsten filament (200 V–30 keV).

The Transmission Electron Microscope (TEM) study was performed using FEI Tecnai G2 model, equipped with a GATAN Tridiem camera and a GATAN Imaging Filter. The field emission gun was operated during the study at 200 kV.

The TEM has been adapted for the examination of highly active or irradiated nuclear materials thanks to a flange that has been inserted in the octagon hosting the objective lenses, and a glove box mounted on this flange around the compustage^30^. The sample was prepared by crushing, as explained in previous work^31^; the so-prepared sample grids were then introduced in a Plasma cleaner machine in order to get rid of any organic residues, and brought to the TEM for the analysis making use of a La Calhène DPTE® system.

Samples were characterized by powder X-ray diffraction on a Rigaku MiniFlex 600 diffractometer with a θ–2θ configuration using Cu Kα1-α2 radiation. The patterns were measured from 15°−120° in 2Θ angle with a scan/step of 0.02° scan/step and a scan speed of 0.29°/min. The software Topas version 5 was used to refine the XRD data.

### Functional properties characterization

A shielded “laser-flash” device designed and constructed at JRC Karlsruhe^32,33^ was used for the measurement of the thermal diffusivity *α* of the samples. The latter are parts of a disk with plane and parallel faces. The thickness of the samples is measured at ambient temperature for different positions at the samples surface in order to verify that the faces are plane and parallel. The effect of thermal dilatation on the thickness is taken into account using the thermal expansion coefficient recommended by Venkata Krishnan *et al*.^34^. The samples are heated under vacuum (10^−5^–10^−6^ Pa of nitrogen) up to the measurement temperature in a high frequency furnace. As soon as a stable temperature is obtained, a laser pulse of 2 ms is applied to the front surface of the disk. The temperature perturbation on the opposite surface is recorded by a pyrometer and the obtained thermograms are analysed in order to deduce α and the heat loss coefficients by a numerical fitting procedure taking into account explicitly the laser pulse length^35^. The precision of measurements is better than 5%, and is mainly determined by the samples thickness variations. The experiments were carried out starting at 500 K with the aim of measuring α at increasing temperatures and of examining recovery effects after laboratory thermal annealing above the fuel irradiation temperature. Thermal cycles were applied corresponding to selected sequences of annealing temperatures (Tann) up to 1550 K.

### Data Availability

The datasets generated and/or analyzed during the current study are available from the corresponding author on reasonable request.
